# CT‐determined low skeletal muscle index predicts poor prognosis in patients with colorectal cancer

**DOI:** 10.1002/cam4.7328

**Published:** 2024-06-24

**Authors:** Yue Feng, Xiao‐Hong Cheng, Mei Xu, Rui Zhao, Qian‐Yi Wan, Wei‐Hua Feng, Hua‐Tian Gan

**Affiliations:** ^1^ Department of Geriatrics and National Clinical Research Center for Geriatrics, West China Hospital Sichuan University Chengdu Sichuan China; ^2^ Division of Gastrointestinal Surgery, Department of General Surgery, West China Hospital Sichuan University Chengdu Sichuan China; ^3^ Department of Gastroenterology and Hepatology, West China Hospital Sichuan University Chengdu Sichuan China; ^4^ Department of Laboratory Medicine, West China Hospital Sichuan University Chengdu Sichuan China; ^5^ Laboratory of Inflammatory Bowel Disease, the Center for Inflammatory Bowel Disease, Clinical Institute of Inflammation and Immunology, Frontiers Science Center for Disease‐Related Molecular Network, West China Hospital Sichuan University Chengdu China

**Keywords:** colorectal cancer, major complications, overall survival, sarcopenia

## Abstract

**Background:**

Sarcopenia is highly prevalent among patients with colorectal cancer (CRC). Computed tomography (CT)‐based assessment of low skeletal muscle index (SMI) is widely used for diagnosing sarcopenia. However, there are conflicting findings on the association between low SMI and overall survival (OS) in CRC patients. The objective of this study was to investigate whether CT‐determined low SMI can serve as a valuable prognostic factor in CRC.

**Methods:**

We collected data from patients with CRC who underwent radical surgery at our institution between June 2020 and November 2021. The SMI at the third lumbar vertebra was calculated using CT scans, and the cutoff values for defining low SMI were determined using receiver operating characteristic curves. Univariate and multivariate analyses were performed to assess the associations between clinical characteristics and postoperative major complications.

**Results:**

A total of 464 patients were included in the study, 229 patients (46.7%) were classified as having low SMI. Patients with low SMI were older and had a lower body mass index (BMI), a higher neutrophil to lymphocyte ratio (NLR), and higher nutritional risk screening 2002 (NRS2002) scores compared to those with normal SMI. Furthermore, patients with sarcopenia had a higher rate of major complications (10.9% vs. 1.3%; *p* < 0.001) and longer length of stay (9.09 ± 4.86 days vs. 8.25 ± 3.12 days; *p* = 0.03). Low SMI and coronary heart disease were identified as independent risk factors for postoperative major complications. Moreover, CRC patients with low SMI had significantly worse OS. Furthermore, the combination of low SMI with older age or TNM stage II + III resulted in the worst OS in each subgroup analysis.

**Conclusions:**

CT‐determined low SMI is associated with poor prognosis in patients with CRC, especially when combined with older age or advanced TNM stage.

## 1 INTRODUCTION

Colorectal cancer (CRC) is the most common cancer in the gastrointestinal tract. It was estimated that over 1.9 million new CRC cases and about 1 million deaths occurred in 2020, and about one in 10 cancer cases and deaths were CRC in the world.^1^ This lethal malignant disease, presents a significant concern as 25% of localized CRC patients later develop metastases, while survival beyond 5 years from new CRC diagnoses is seen in less than 20% of patients.^2^ CRC patients face an elevated risk of malnutrition or even cachexia throughout every stage of the disease, which leads to the progressive loss of lean body mass, including skeletal muscle.^3^

Sarcopenia is a common skeletal muscle disorder that involves the loss of muscle mass and function,^4^, ^5^ and it was first recognized as an independent disease with an ICD‐10 code in 2016.^6^ Recent research has indeed highlighted sarcopenia as a new index for estimating prognosis in many diseases, particularly in elderly individuals. An umbrella review of meta‐analyses conducted by Xia et al. examined the association between sarcopenia and various adverse health‐related outcomes.^7^ The review found that sarcopenia significantly increased the risk of multiple adverse health‐related outcomes, including mortality, functional decline, falls, and hospitalization. These associations were particularly prominent in elderly individuals. Meanwhile, multiple studies have demonstrated a significant correlation between sarcopenia and unfavorable outcomes in cancer patients, including those diagnosed with lung cancer, gastrointestinal cancer, as well as head and neck cancer.^8^, ^9^, ^10^ For example, in patients with CRC, reduced skeletal muscle mass, a characteristic of sarcopenia, has been identified as a predictive factor for an unfavorable prognosis.^11^ On the other hand, sarcopenia exhibits a substantial prevalence ranging from 38.8% to 59.8% in individuals with CRC, and has been strongly linked to adverse postoperative outcomes.^12^, ^13^, ^14^, ^15^

The current diagnostic criteria of sarcopenia were depended on a comprehensive assessment including muscle mass, muscle strength, and physical performance.^4^ A single slice of the level of the third lumbar vertebra in CT scans has been widely intercepted in the analysis of body tissues, especially to help with the diagnosis of sarcopenia, while abdominal CT is routinely performed for preoperative assessment in cancer patients. Therefore, many studies examined the impact of CT‐determined low skeletal muscle index (SMI) instead of a complete definition of sarcopenia.

Although some studies have indicated a correlation between skeletal muscle mass and aspects such as mortality rate, and prognosis, in CRC, there is limited research on the relationship between skeletal muscle mass and the occurrence of postoperative complications in CRC, and consequently, the conclusions remain uncertain.^16^, ^17^, ^18^, ^19^, ^20^, ^21^ In this study, we aimed at investigating the associations between low SMI and complications and analyze the impact of low SMI on the multiple clinicopathological variables of CRC patients in our hospital.

## 2 METHODS

### 2.1 Patients and data collection

In this retrospective study, we enrolled patients with CRC treated at the Division of Gastrointestinal Surgery, West China Hospital, Sichuan University, from June 2020 to November 2021. The inclusion criteria were as follows: (a) adult patients undergoing radical surgery; (b) confirmation of CRC diagnosis by pathology; (c) completion of abdominal CT scan at our hospital before surgery; (d) no history of other malignant tumors. The exclusion criteria were as follows: (a) Patients who underwent non‐radical surgery. (b) Patients who underwent emergency surgery due to acute complications like obstruction, perforation, and enterorrhagid and so forth. (c) Patients lost to follow‐up were excluded from the study.

The data for this study were obtained from the medical records and examination reports of the patients. Neutrophil to lymphocyte ratio (NLR) was assessed as the systemic inflammatory response. Upon admission, the nutritional risk of each patient was assessed using the Nutritional Risk Screening 2002 (NRS2002) by a nurse. Postoperative major complications were defined as surgical complications of Grade III or higher.^22^ Patients were followed up either at an outpatient clinic or through telephone communication. The latest follow‐up data were collected in May 2022. The ethics committee of West China Hospital approved this study on 26th, October 2021, and the registration number is 2021 (1195). The study was conducted in accordance with the principles of the Declaration of Helsinki. The requirement for informed consent was exempted due to the retrospective nature of the study. Patient information was anonymized and de‐identified prior to analysis.

### 2.2 Assessment of skeletal muscle mass

The skeletal muscle mass of each patient was assessed based on the preoperative abdominal CT images of the third lumbar vertebra (L3) level. The skeletal muscle area (cm^2^) of the L3 level was semi‐automatically analyzed using the software of BMI_CT (Seoul, South Korea).^23^ Particularly, for skeletal muscle, the Hounsfield Unit (HU) threshold was set as −29 to 150.^24^, ^25^ The quantity of skeletal muscle mass was represented by the SMI of the L3 level, which was reported as total skeletal muscle area (cm^2^) of L3/height squared (m^2^).^26^, ^27^

### 2.3 Statistical analysis

Continuous variables were presented as mean (± standard deviation) or median with range. Data comparison was performed using the *t*‐test or Mann–Whitney *U*‐test. Categorical data were described as the number of cases, with data comparison conducted using the chi‐squared test or Fisher's exact test. Two‐sided *p*‐values were utilized in this study, with a *p*‐value of <0.05 considered statistically significant. Univariate logistic regression analysis was employed to investigate the associations between various clinical characteristics and postoperative major complications. Characteristics with a *p*‐value of <0.1 in the univariate analysis were further examined in multivariate analysis. Survival analysis was carried out using Kaplan–Meier survival curves and analyzed using log‐rank tests. All statistical analyses were performed using R 4.3 and GraphPad Prism version 8.0.

## 3 RESULTS

**FIGURE 1:**
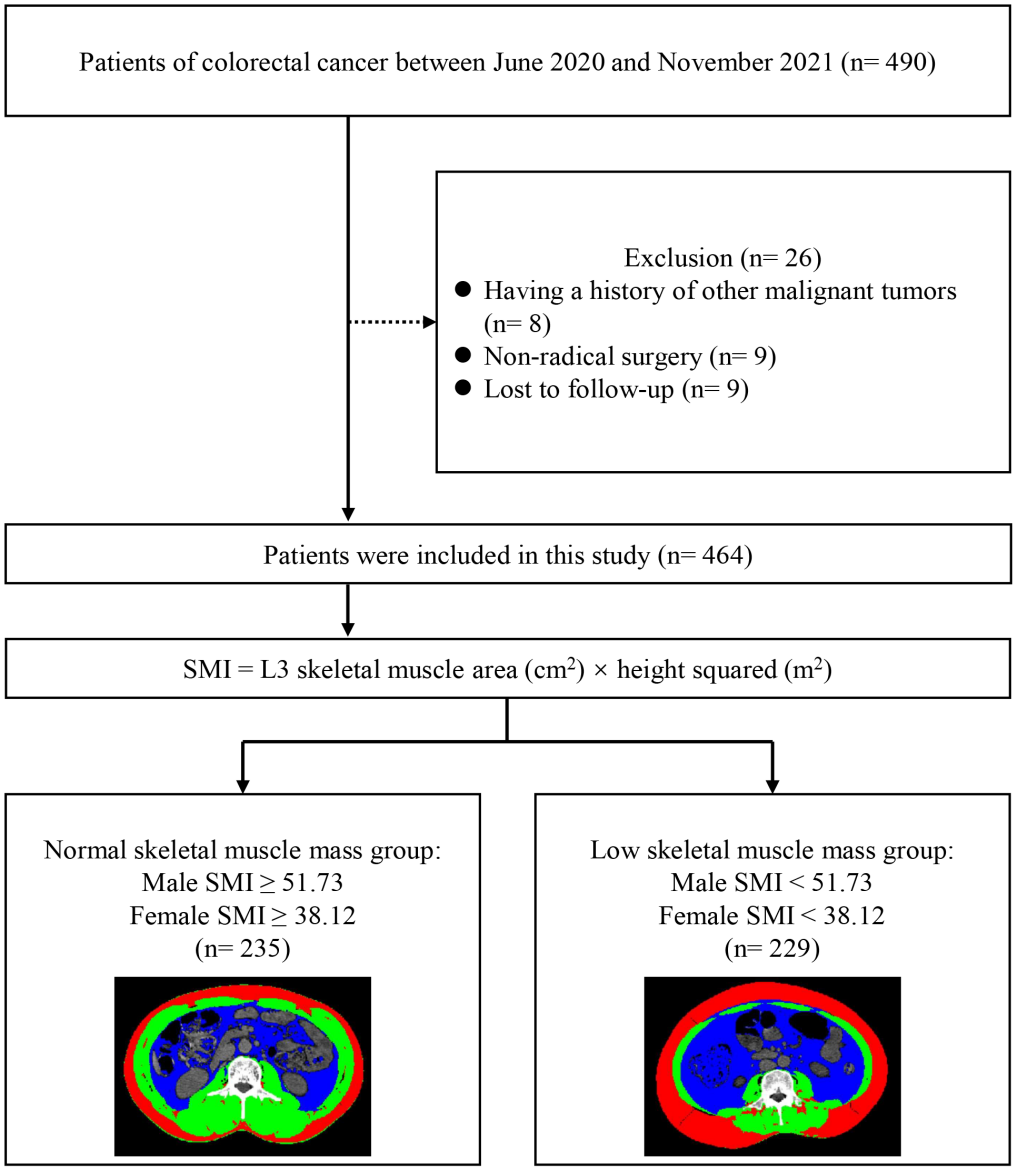
Flow diagram of patients.

In this study, we identified 490 potentially eligible patients between June 2020 and November 2021. All the patients underwent open surgery. However, 26 patients were excluded based on the established exclusion criteria (Figure 1). Among the 464 patients included in the study, there were 283 male patients and 181 female patients. Among these patients, 28 experienced major surgical complications (Table S1). The preoperative SMI of each patient was calculated by analyzing the skeletal muscle area at the L3 level. Figure 1 displays representative CT images of the skeletal muscle mass from both the normal and low SMI groups.

**FIGURE 2:**
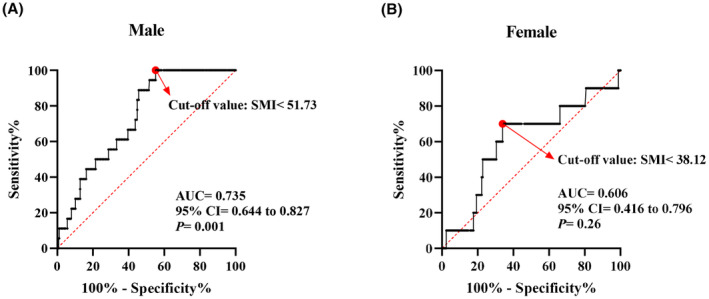
ROC curves of low SMM between (A) Male and (B) Female for the detection of major complications.

To determine the cut‐off values of SMI for defining low skeletal muscle mass, ROC curves were constructed for male and female patients separately, with major surgical complications as the outcome variable. Using Youden's index, a cutoff of SMI ≤51.73 cm^2^/m^2^ was identified for male patients, while a cutoff of SMI ≤38.12 cm^2^/m^2^ was determined for female patients. Patients with SMI values below these cutoffs were classified as having low skeletal muscle mass (Figure 2). The patients were categorized into two groups based on the criteria: the low skeletal muscle index (low SMI) group and the normal skeletal muscle index (normal SMI) group.

Among the 464 patients included in the study, 229 patients (49.4%) were classified as having low SMI. Male patients exhibited a significantly higher rate of low SMI compared to females (71.6% in males vs. 28.4% in females; *p* < 0.001) (Table 1). Furthermore, patients with low SMI were found to be significantly older than those with normal SMI (62.72 ± 11.85 years in low SMI vs. 59.11 ± 11.05 years in normal SMI; *p* < 0.001). Additionally, low SMI was associated with a significantly lower BMI (24.78 ± 2.67 in low SMM vs. 21.29 ± 2.66 in normal SMI; *p* < 0.001), a higher neutrophil to lymphocyte ratio (NLR) (2.35 ± 1.39 in low SMM vs. 2.67 ± 1.82 in normal SMI; *p* = 0.03), and a higher rate of NRS2002 score ≥3 (85.2% in low SMI vs. 73.2% in normal SMI; *p* = 0.002) (Table 1). The prevalence of low SMI did not significantly differ between colon cancer and rectal cancer or among different TNM stages. In addition, the presence of low SMI was not significantly associated with cigarette smoking, alcohol drinking, hypertension, coronary heart disease, and diabetes (Table 1). Regarding surgery‐related outcomes, it was observed that patients with low SMI had a significantly higher rate of major complications compared to those with normal SMI (10.9% in low SMI vs. 1.3% in normal SMI; *p* < 0.001). Additionally, patients with low SMI had a slightly longer length of stay compared to those with normal SMI (8.25 ± 3.12 days in low SMI vs. 8.09 ± 4.86 days in normal SMI; *p* = 0.03) (Table 1).

**TABLE 1:** Clinical characteristics between patients with normal and low SMI.

Characteristics	Normal SMI (*n* = 235)	Low SMI (*n* = 229)	*p* value
Male/female, *n*	119/116	164/65	<0.001
Age, mean ± SD (years)	59.11 ± 11.05	62.72 ± 11.85	<0.001
SMI, mean ± SD			
Male	56.66 ± 4.52	45.38 ± 4.54	<0.0001
Female	44.28 ± 4.15	34.91 ± 2.47	<0.0001
BMI, mean ± SD	24.78 ± 2.67	21.29 ± 2.66	<0.0001
NLR, mean ± SD	2.35 ± 1.39	2.67 ± 1.82	0.03
NRS2002			
<2	63	34	0.002
≥3	172	195	
Tumor site			
Colon cancer	89	91	0.68
Rectal cancer	146	138	
TNM stage, *n*			
I	56	55	0.99
II	86	84	
III	93	90	
Postoperative adjuvant chemotherapy, *n* (yes/no)	168/67	155/74	0.37
Cigarette smoking, *n* (yes/no)	35/200	47/182	0.11
Alcohol drinking, *n* (yes/no)	24/211	24/205	0.93
Hypertension, *n* (yes/no)	47/188	43/186	0.74
Coronary heart disease, *n* (yes/no)	10/225	9/220	0.86
Diabetes, *n* (yes/no)	24/211	27/202	0.59
Major complication, *n* (yes/no)	3/232	25/204	<0.0001
Length of stay, mean ± SD (days)	8.25 ± 3.12	9.09 ± 4.86	0.03

Abbreviations: BMI, body mass index; NLR, neutrophil to lymphocyte ratio; NRS2002, nutritional risk screening 2002; SD, standard deviation; SMI, skeletal muscle index; SMM, skeletal muscle mass; TNM stage, tumor‐node‐metastasis stage.

To assess the potential associations between SMI and the risk of postoperative major complications, logistic regression analysis was performed. Both univariate and multivariate analyses indicated that low SMI (multivariate analysis: HR 8.75, 95% CI 2.56 to 29.91; *p* = 0.001) and coronary heart disease (multivariate analysis: HR 5.05, 95% CI 1.34 to 18.95; *p* = 0.002) were identified as independent risk factors for postoperative major complications (Table 2).

**TABLE 2:** Univariate and multivariate analysis of major complications.

Characteristics	Univariate		Multivariate	
	HR (95% CI)	*p*	HR (95% CI)	*p*
Age ≥ 60 years, (<60 as ref)	2.10 (0.91 to 4.87)	0.08	1.43 (0.59 to 3.46)	0.43
Female, (male as ref)	1.16 (0.52 to 2.58)	0.71		
BMI ≥20, (<20 as ref)	0.54 (0.23 to 1.26)	0.15		
NLR ≥3, (<3 as ref)	1.55 (0.68 to 3.53)	0.30		
Low SMI, (normal SMI as ref)	9.48 (2.82 to 31.85)	<0.0001	8.75 (2.56 to 29.91)	0.001
NRS2002 ≥ 3, (<3 as ref)	3.62 (0.84 to 15.53)	0.08	2.62 (0.59 to 11.64)	0.21
Tumor site, (colon cancer as ref)	1.15 (0.52 to 2.55)	0.73		
TNM stage II + III, (stage I as ref)	1.95 (0.66 to 5.75)	0.23		
Cigarette smoking, (no as ref)	1.01 (0.37 to 2.75)	0.98		
Alcohol drinking, (no as ref)	0.65 (0.15 to 2.84)	0.57		
Hypertension, (no as ref)	1.42 (0.58 to 3.45)	0.44		
Diabetes, (no as ref)	1.84 (0.67 to 5.08)	0.24		
Coronary heart disease, (no as ref)	4.68 (1.44 to 15.18)	0.01	5.05 (1.34 to 18.95)	0.02

Abbreviations: BMI, body mass index; HR, hazard ratios; NLR, neutrophil to lymphocyte ratio; NRS2002, nutritional risk screening 2002; SD, standard deviation; SMM, skeletal muscle mass; TNM stage, tumor‐node‐metastasis stage; 95% CI, 95% confidence intervals.

**FIGURE 3:**
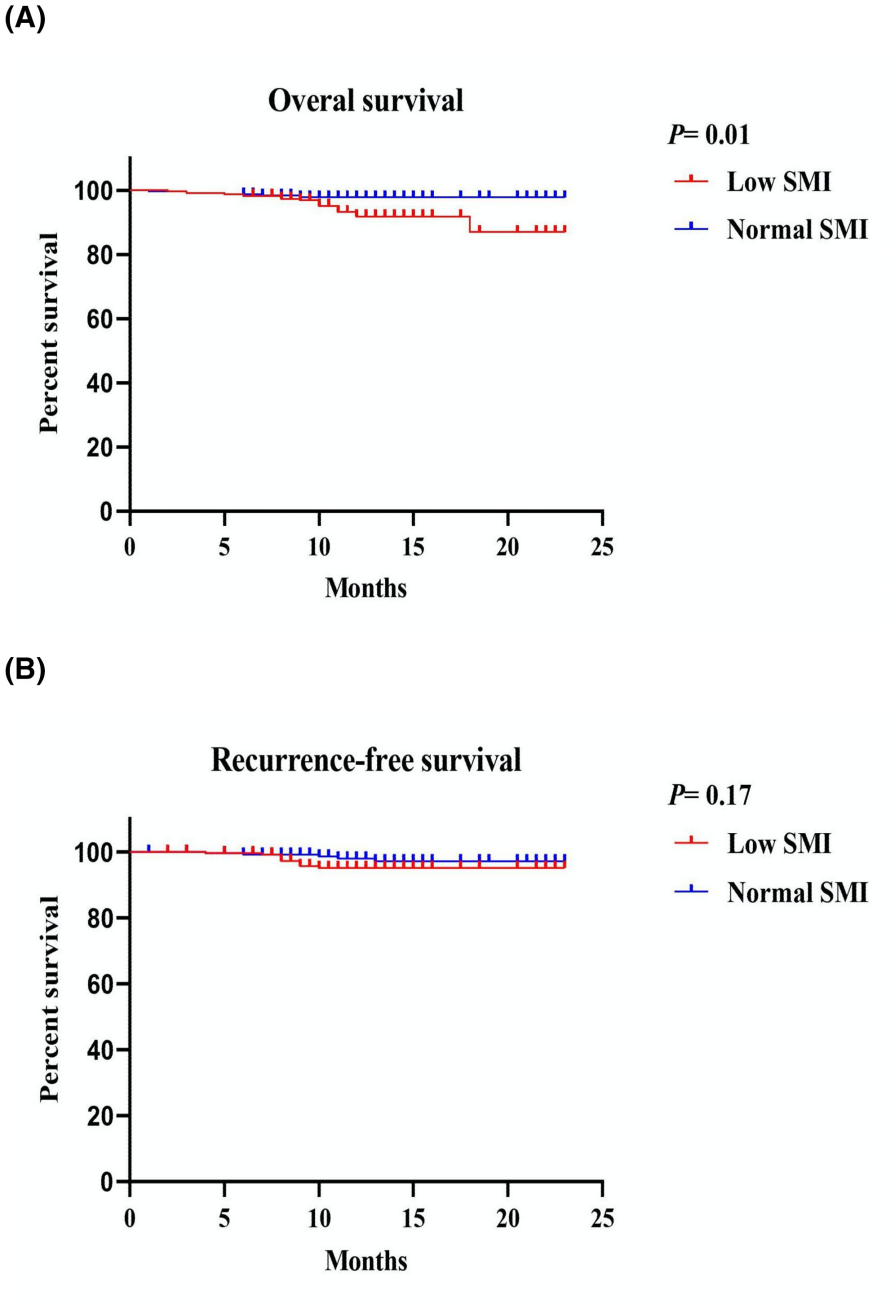
Kaplan–Meier survival curves for the associations between SMM and (A) overall survival (OS); (B) recurrence free survival.

To determine the impact of low SMI on the survival of CRC patients, Kaplan–Meier survival analyses were performed for overall survival (OS) and recurrence‐free survival (RFS). The results revealed that patients with low SMI had a significantly worse OS compared to those with normal SMI (*p* = 0.01, Figure 3). However, there were no significant differences observed in RFS between patients with low SMI and normal SMI (*p* = 0.17, Figure 3).

**FIGURE 4:**
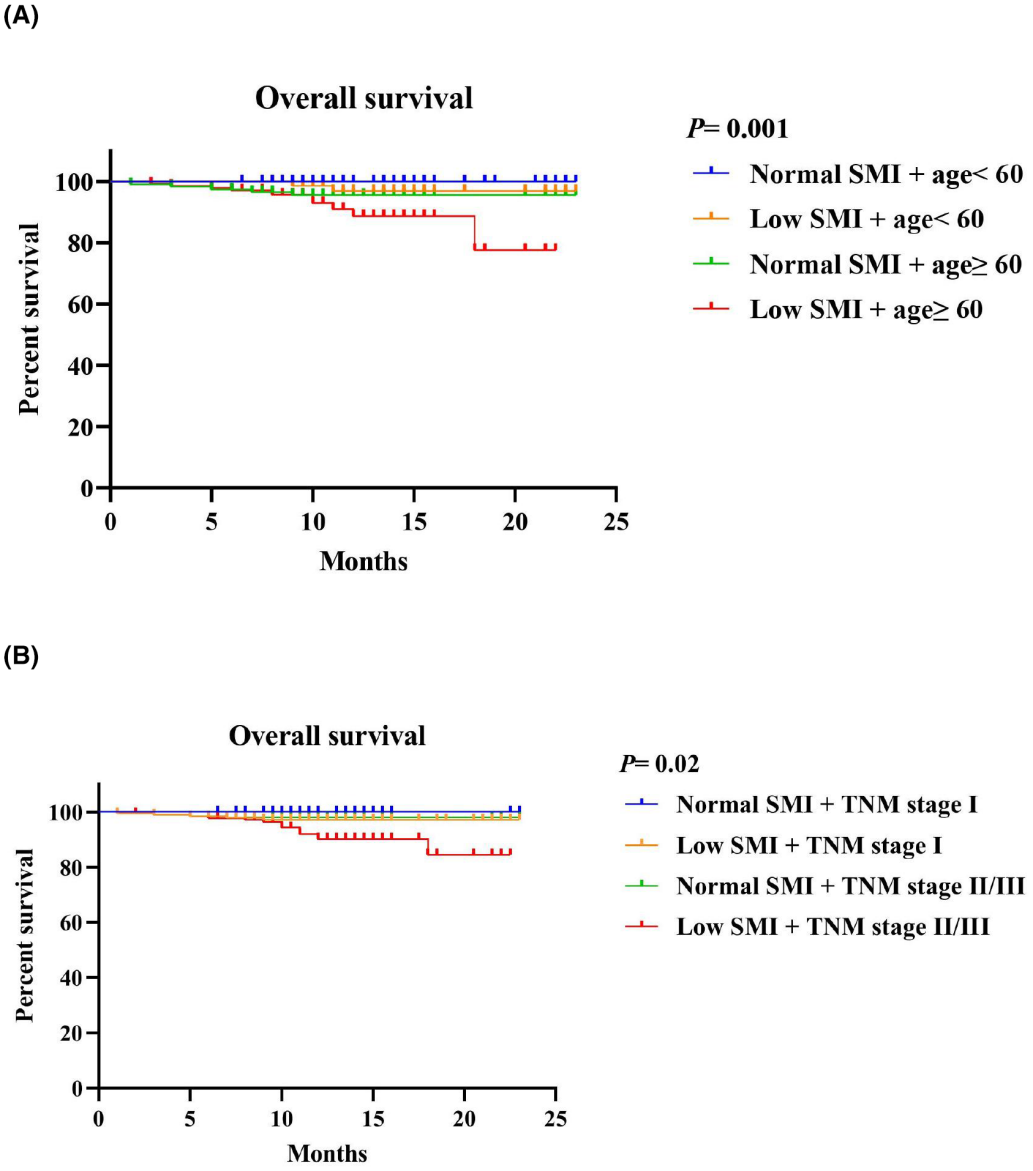
Survival analysis for the combination of SMM and (A) age; (B) TNM stage.

Furthermore, when considering the combination of SMI, TNM stage, and age, we observed the following patterns in OS: (1) Comparing patients with normal SMI and age < 60 to different subgroups: Patients with low SMI and age ≥ 60 had worse OS (*p* = 0.001). Patients with normal SMI and age ≥ 60 showed better OS than those with low SMI and age < 60. (2) Examining the combination of SMI and TNM stage: Patients with low SMI and TNM stage II/III exhibited the worst OS (*p* = 0.02), compared to those with normal SMI and TNM stage I. Following this pattern, patients with low SMI and TNM stage I, as well as those with normal SMI and TNM stage II/III, demonstrated progressively better OS. These findings highlight the significance of considering the combined effects of SMI, TNM stage, and age on the overall prognosis of patients (Figure 4).

## 4 DISCUSSION

In this study involving 464 patients with CRC, we made several important observations regarding the association of various factors with low SMI and its impact on patient outcomes. First, we found that male gender, older age, lower body mass index (BMI), higher NLR, higher NRS2002 score, and poor postoperative outcomes (such as a higher rate of major complications and longer hospital stays) were significantly associated with a higher likelihood of having low SMI. Second, logistic regression analysis indicated that low SMI and the presence of coronary heart disease were the only factors significantly associated with major complications in these patients. Furthermore, we discovered that patients with low SMI had a significantly worse OS compared to those with normal SMI. However, there were no significant differences observed in RFS between patients with low SMI and normal SMI. Additionally, when considering the combination of SMI and age or TNM stage in subgroup analyses, we identified significant differences in OS. These findings suggest that low SMI is associated with poor prognosis and increased risk of major complications in patients with CRC. Considering SMI in combination with age and TNM stage may provide further insights into patient outcomes.

Skeletal muscle tissues serve as substantial metabolic organs in the human body. The loss of skeletal muscle mass can be attributed to various factors. Inadequate energy intake, along with systemic diseases like cancer, is widely acknowledged as common causes of muscle loss. A deficiency in energy intake, whether due to poor nutrition or reduced dietary consumption, can result in muscle wasting.^28^ In the case of systemic diseases such as cancer, both the disease itself and its treatments can contribute to muscle mass decline.^5^ These factors highlight the importance of addressing energy intake and managing systemic diseases to prevent and mitigate muscle loss. One possible mechanism that may link reduced skeletal muscle mass and CRC involves the upregulation of tumor‐induced inflammatory cytokines. These cytokines, such as tumor necrosis factor alpha (TNF‐α) and interleukin 6 (IL‐6), can have detrimental effects on muscle tissue.^29^ First, the elevated levels of TNF‐α and IL‐6 in the presence of CRC can lead to insulin resistance, making it harder for muscle cells to utilize glucose effectively. Insulin resistance impairs the anabolic effects of insulin on muscle tissue, resulting in decreased protein synthesis and muscle growth.^30^ Additionally, the increased inflammatory cytokines can promote muscle protein degradation and trigger the catabolism of myofibers.^29^ This accelerated breakdown of muscle proteins contributes to the loss of skeletal muscle mass. Furthermore, chronic inflammation induced by CRC can disrupt the normal balance between muscle protein synthesis and breakdown, favoring catabolic processes. This imbalance further exacerbates muscle wasting.^31^ It is important to note that these mechanisms are complex and interconnected, involving various signaling pathways and cellular processes. The upregulation of tumor‐induced inflammatory cytokines in CRC contributes to insulin resistance, promotes muscle protein degradation, and disrupts muscle homeostasis, ultimately leading to the loss of skeletal muscle mass.^32^ Reduced muscle mass also can have significant implications on drug distribution and may even increase the risk of toxicity in patients undergoing chemotherapy. This, in turn, can lead to a poor prognosis for cancer patients. Muscle mass plays a critical role in the metabolism and distribution of drugs within the body. It serves as a reservoir for medications and aids in their clearance. However, when there is a decrease in muscle mass, the capacity for drug storage and metabolism is compromised, resulting in altered drug pharmacokinetics.^33^ This can lead to higher drug concentrations, prolonged exposure to the drugs, and an increased risk of toxicity as the body struggles to effectively process and eliminate them. The heightened toxicity associated with reduced muscle mass can pose significant challenges for patients receiving chemotherapy. It can increase the occurrence of adverse effects and complications during treatment. For instance, bone marrow suppression, gastrointestinal toxicity, and compromised immune function can all be amplified, impacting treatment outcomes and overall prognosis.^34^, ^35^, ^36^ Moreover, decreased muscle mass is often accompanied by physical weakness and functional limitations. This can result in reduced tolerance for chemotherapy, heightened treatment‐related side effects, and a higher likelihood of treatment interruptions or dose reductions. These factors can compromise the effectiveness of chemotherapy and contribute to a poor prognosis in individuals with cancer. Therefore, it is crucial to address and manage reduced muscle mass in cancer patients. Implementing interventions such as nutritional support and appropriate physical activity can help optimize drug distribution, minimize treatment toxicity, and ultimately improve prognosis.^37^, ^38^

Our study indicated a strong connection between low SMI and OS of CRC patients. Several studies tried to find the mechanism. Low skeletal muscle status may affect immune system in many aspects.^37^ Few studies investigated the association between skeletal muscle and T‐cell subsets in cancer patients. We noticed a study of extrahepatic cholangiocarcinoma reporting that patients with sarcopenia were associated with lower tumor‐infiltrating CD8+ T cells.^39^ Tumor‐infiltrating CD8+ T cells are key immune cells that directly fight tumor cells. Several studies have suggested that a higher level of tumor‐infiltrating CD8+ T cells was associated with improved survival in multiple cancer patients including CRC.^40^, ^41^, ^42^, ^43^ Meanwhile, skeletal muscle in humans is one of the centers of systematic metabolism which makes up about 40% of total body weight and includes more than 50% of all proteins.^44^ As a result of reduced muscle mass, except weight loss, malnutrition, or cachexia are also major complications. The bad physical condition caused by malnutrition cachexia in return worsens the body's ability to withstand cancer attack, then leads to disease progression.^45^ These may partly explain the combination of low SMI and late TNM stages predicted worst OS in CRC patients. We found that low SMI patients of older age also had bad OS. Age is recognized as an important cause of muscle loss. After the age of 50, people could lose an average of over 15% of their skeletal muscle mass per decade.^46^ Considering that age is primarily a crucial clinicopathological variable to predict survival, the combination of low SMI and older age could more accurately distinguish patients with different prognoses.

CT is an important tool for measuring body composition, and the skeletal muscle area of L3 level can reflect the total skeletal muscle of the human body.^4^ One recent umbrella review also indicated that CT was the majority tool used for assessing skeletal muscle mass in sarcopenia‐related clinical studies.^7^ In this study, we analyzed the preoperative abdominal CT image and calculated the SMI of L3 level for each included patient. Through ROC curves and Youden's index, we identified the sex‐specific cut‐off values of SMI for defining sarcopenia (51.73 cm^2^/m^2^ for males and 38.12 cm^2^/m^2^ for females), which are close to another widely applied cut‐off values for defining sarcopenia in cancer patients (52.4 cm^2^/m^2^ for males and 38.5 cm^2^/m^2^ for females).^47^, ^48^, ^49^ It was reported that preoperative low SMI was significantly associated with increased postoperative complications in cancer patients.^12^ In our study, we discovered that the SMI exhibited superior diagnostic capabilities in detecting major surgical complications compared to other factors, such as the NRS2002 score, BMI, age, and the presence of coronary disease. Additionally, patients with low SMI were found to have a significantly higher rate of prolonged length of hospital stay. Many research and our study also has shown that the measurement of skeletal muscle composition using CT scans has a high predictive value for predicting postoperative complications and prognosis, and thus, this indicator is crucial for clinicians.^11^, ^25^, ^50^ CT scans provide detailed information about skeletal muscle, which can be used to assess a patient's overall physical condition and muscle function. Prior to surgery, CT scans can help identify the risk of low skeletal muscle mass in patients, enabling appropriate measures to be taken to reduce the occurrence of complications. Low SMI is associated with increased surgical risks and adverse outcomes, including longer hospital stays.^35^ Accurate measurement and evaluation of skeletal muscle composition allow healthcare professionals to better predict surgical outcomes and prognosis, and tailor individualized treatment and rehabilitation plans as needed. Therefore, it is of utmost importance for clinicians to give due attention to the use of CT scans for measuring skeletal muscle composition. This indicator provides valuable guidance for clinical decision‐making and postoperative management, assisting in improving patient outcomes and reducing the risk of complications. Further research and clinical application will help validate and promote the effectiveness and significance of this approach.

There were several limitations in this study. First, the shortage of CT for evaluating sarcopenia is that CT can only assess skeletal muscle mass instead of muscle strength and physical function. Therefore, CT‐determined sarcopenia is only characterized by a low SMI. Because of the lack of data about the assessment of muscle strength and physical performance in this retrospective study, further prospective studies with a complete assessment of sarcopenia are required in the future. Last, our study's sample is somewhat small, and further research on this topic with a larger sample size is still necessary to confirm our findings.

In conclusion, the use of CT to determine low SMI is a reliable parameter for predicting unfavorable postoperative outcomes in patients with CRC. Additionally, there is a significant correlation between low SMI and worse OS in CRC patients. However, it is important to acknowledge the limitations of this study and recognize the need for further prospective research on this topic in the future.

## AUTHOR CONTRIBUTIONS

**Hua‐Tian Gan:** Supervision (equal); visualization (equal). **Yue Feng:** Formal analysis (equal); methodology (equal); writing – original draft (equal). **Xiao‐Hong Cheng:** Investigation (equal); software (equal); writing – original draft (equal). **Mei Xu:** Conceptualization (equal); data curation (equal); writing – original draft (equal). **Wei‐Hua Feng:** Conceptualization (equal); project administration (equal); resources (equal). **Rui Zhao:** Methodology (equal); software (equal); writing – review and editing (equal). **Qian‐Yi Wan:** Investigation (equal); resources (equal); writing – review and editing (equal).

## FUNDING INFORMATION

This work was supported by the National Natural Science Foundation of China (no.82070560), and 1.3.5 project for disciplines of excellence, West China Hospital, Sichuan University (no. ZYGD18023).

## CONFLICT OF INTEREST STATEMENT

There was no conflict of interest.

## ETHICS STATEMENT

The study was approved by the ethics committee of West China Hospital, Sichuan University.

## Supporting information


**Table S1.** Classification of major complications.

## Data Availability

All data generated or analyzed during this study are included in this published article.
