# Supplementary material for: CT‐determined low skeletal muscle index predicts poor prognosis in patients with colorectal cancer
Source: Cancer Med. 2024 Jun 24;13(12):e7328. doi: 10.1002/cam4.7328 (PMC11196831; doi:10.1002/cam4.7328)
Supplement: Supplementary file 1 — Table S1. Classification of major complications. [file CAM4-13-e7328-s001.docx]

Supplementary Table 1. Classification of major complications.

| Characteristics | Patients(n=28) |
| --- | --- |
| Anastomotic leak | 22(78.6) |
| Postoperative infection | 10(35.7) |
| Enterorrhagid | 3(10.7) |
